# Efficacy and safety of duloxetine versus placebo in adolescents with juvenile fibromyalgia: results from a randomized controlled trial

**DOI:** 10.1186/s12969-019-0325-6

**Published:** 2019-05-28

**Authors:** Himanshu P. Upadhyaya, Lesley M. Arnold, Karla Alaka, Meihua Qiao, David Williams, Renata Mehta

**Affiliations:** 1 Eli Lilly and Company, Lilly Corporate Center, Indianapolis, IN 46285 USA; 2 University of Cincinnati College of Medicine, Cincinnati, OH USA; 3 Syneos Health, Raleigh, NC USA; 4 Focus Clinical Consulting Inc, Toronto, ON Canada

**Keywords:** Juvenile fibromyalgia, Duloxetine, Serotonin and norepinephrine reuptake inhibitor

## Abstract

**Background:**

Currently, there are no medications approved for the treatment of juvenile fibromyalgia (JFM). We evaluated the safety and efficacy of duloxetine 30/60 mg once daily (QD) versus placebo in adolescents with JFM.

**Methods:**

In this Phase 3b, multisite (US, Argentina, Puerto Rico, and India) trial, patients aged 13–17 years with JFM and a score of ≥4 on the Brief Pain Inventory-Modified Short Form: Adolescent Version (BPI) 24-h average pain severity score were randomized to duloxetine or placebo for the 13-week double-blind period. The starting duloxetine dose was 30 mg, with a target dose of 60 mg QD, as tolerated. The primary endpoint was the mean change in 24-h average pain severity of the Brief Pain Inventory (BPI) from baseline to Week 13, analyzed using mixed-model repeated measures (MMRM) technique. Secondary measures were BPI severity and interference scores; treatment response (≥30%, ≥50% reductions on BPI average pain severity); Pediatric Pain Questionnaire; Clinical Global Impression of Severity: Overall and Mental Illness scales; Functional Disability Inventory: child and parent versions; Children’s Depression Inventory; Multidimensional Anxiety Scale for Children; and safety and tolerability. Continuous secondary efficacy measures were analyzed using analysis of covariance or MMRM, and categorical data using Cochran-Mantel-Haenszel test and Fisher’s exact test, where appropriate.

**Results:**

A total of 184 patients with JFM received duloxetine (*N* = 91) or placebo (*N* = 93), of which 149 patients (81.0%) completed the 13-week double-blind treatment period. Baseline characteristics were comparable between groups; majority of the patients were Caucasian (77.17%) and females (75.0%), with a mean age of 15.53 years. For the primary measure, BPI average pain severity, the mean change was not statistically different between duloxetine and placebo (− 1.62 vs. -0.97, respectively; *p* = .052). For secondary efficacy outcomes, statistically significantly more duloxetine- versus placebo-treated patients had a treatment response (≥30% and ≥50% reductions on BPI average pain severity) and improvement of the general activity and relationships items on the BPI interference subscale. The percentage of patients reporting at least 1 treatment-emergent adverse event was higher in the duloxetine versus placebo groups (82.42% vs. 62.37%, respectively; *p* = .003). The overall safety profile of duloxetine in this study was similar to that reported previously in duloxetine pediatric trials of other indications.

**Conclusions:**

The primary study outcome, mean change in 24-h BPI average pain severity rating from baseline to Week 13, did not significantly improve with duloxetine compared to placebo in patients with JFM. However, significantly more patients on duloxetine compared to placebo had a ≥30% and ≥50% reduction in pain severity. There were no new safety concerns related to duloxetine in the study population.

**Trial registration:**

ClinicalTrials.gov Identifier: NCT01237587. Registered 08 November, /2010.

**Electronic supplementary material:**

The online version of this article (10.1186/s12969-019-0325-6) contains supplementary material, which is available to authorized users.

## Background

Chronic pain affects approximately 25% of school-aged children [1, 2]. Juvenile-onset fibromyalgia, commonly referred to as juvenile fibromyalgia (JFM), affects 2.1–6.1% of school children, mostly adolescent girls, and patients with JFM typically present to pediatric rheumatology clinics for evaluation and treatment [3, 4]. However, the prevalence of JFM in the United States (US) general adolescent population is unknown.

Adolescents with JFM commonly report chronic widespread pain and other associated symptoms that are observed in adults with fibromyalgia (FM) [5]. JFM can have a significantly greater impact on functional disability and school attendance in adolescents, compared to other rheumatic diseases such as juvenile idiopathic arthritis or lupus [6]. Yunus and Masi proposed the first criteria for JFM in 1985 that included chronic widespread pain, the presence of tender points on examination, and associated symptoms such as fatigue and poor sleep [7].

Currently, there are no medications approved for the treatment of JFM in adolescents. Duloxetine, a serotonin and norepinephrine reuptake inhibitor, is approved by the US Food and Drug Administration (FDA) for the treatment of FM, diabetic peripheral neuropathic pain, chronic musculoskeletal pain, major depressive disorder (MDD), and generalized anxiety disorder in adults. This study was conducted to evaluate the safety and efficacy of duloxetine 30/60 mg once daily (QD) compared to placebo in adolescents with JFM.

## Methods

### Study design and study population

**Fig. 1:**
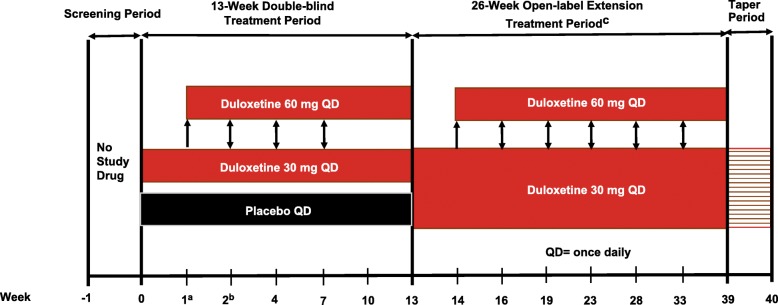
Study Design. **a**. Duloxetine initiated at 30 mg QD dose. At Weeks 1–7, the dose could be increased to 60 mg QD based on investigator’s clinical judgement. If subsequent dose decrease to 30 mg QD was needed, it could not be increased again during the double-blind period. **b**. From Week 2 to 13, patients randomized to placebo or duloxetine 30 mg who discontinued prior to entering the open-label extension treatment period received placebo in the drug taper period. **c**. From Week 14 to 33, dose increases were permitted at scheduled visits (to a maximum dose of 60 mg QD) and dose decreases were permitted at scheduled or unscheduled visits (to a minimum dose of 30 mg QD) based on investigator’s clinical judgement. **d**. Patients on duloxetine 60 mg QD dose were tapered to 30 mg QD, and those who were on 30 mg QD during open label treatment period were not required to enter the drug taper period

This was a Phase 3b, randomized, double-blind, placebo-controlled, multi-site study (ClinicalTrials.gov Identifier: NCT01237587; Fig. 1). Male or Female patients (Argentina = 38, India = 13, Puerto Rico = 5, US = 128) aged 13–17 years with JFM as defined by Yunus and Masi (1985) [7] and pain scores ≥4 on the average pain severity item of the Brief Pain Inventory (BPI) Modified Short Form: Adolescent Version were included. Patients who were previously (within 6 months) treated with duloxetine, were hypersensitive to duloxetine or any inactive ingredients, or had frequent or severe allergic reactions to multiple medications were excluded from the study. Patients taking any excluded medications (e.g., stimulants, antidepressants) that could not be discontinued at Visit 1 (Study period 1 screening phase, which included a medication wash-out period if needed) were excluded. Additionally, patients who had evidence of rheumatologic disorder or had a current diagnosis of juvenile idiopathic arthritis, inflammatory arthritis, or infectious arthritis, or an autoimmune disease (for example, systemic lupus erythematosus), Diagnostic and Statistical Manual of Mental Disorders 4th edition (DSM-IV) Axis I condition or DSM-IV Axis II disorder (investigator judgment) were also excluded with the exception of MDD, generalized anxiety disorder, adjustment disorder, or specific phobias which were allowed based on the investigator’s judgment. The complete list of inclusion and exclusion criteria are included as Additional file 1: Table S1. At the randomization visit (Visit 2), patients who met entry criteria in study period I were randomized in a 1:1 ratio to duloxetine or placebo by a computer-generated random sequence using an interactive voice response system (IVRS), and patients were allowed to take the study drug the morning after Visit 2.

Study protocols and informed consent forms were approved by an Institutional Review Board at each site. The parent/legal representative signed the informed consent form, and the patient signed the assent document (if applicable) prior to initiation of any study-related procedures.

### Study treatment

During the double-blind treatment period, patients randomly received a target dose of 60 mg (2 of the 30 mg capsules) duloxetine or matching placebo QD (Weeks 1–13). Patients randomized to duloxetine received 30 mg QD for 1 week and the dose was then increased to 60 mg (2 of the 30 mg capsules) QD; however, investigators could decrease their dose to 30 mg QD during double-blind treatment period, based on patient’s response. No further changes in the dose were allowed after Week 7, and once the dose was decreased to 30 mg, it could not be increased during the double-blind treatment period. Dose adjustments were performed through dispensing the appropriate investigational product packaging at the site. All dosing modifications during double-blind treatment period were implemented in a blinded manner using IVRS. After 13 weeks of treatment, all patients including those receiving placebo entered a 26-week open-label extension phase (Weeks 14–39), wherein patients received 30 mg duloxetine QD for 1 week and the dose was then increased to 60 mg QD. Moreover, patients could decrease their dose to 30 mg QD during open-label extension phase. Patients who discontinued any time after receiving treatment for 2 weeks in double-blind treatment period or discontinued/completed on 60 mg duloxetine in open-label extension entered a 1-week taper period with 30 mg or placebo, to minimize any discontinuation-emergent adverse events (AEs; Fig. 1). Patients who discontinued on 30 mg duloxetine dose did not receive a 1 week taper period.

### Study assessments

The primary efficacy objective was to compare mean change in 24-h average pain severity of BPI [8] from baseline to Week 13 between duloxetine 30/60 mg QD and placebo. The secondary endpoints were improvement in the following measures at the end of double-blind treatment period (Week 13) and open-label extension (Weeks 14–39): BPI-modified short form: adolescent version severity (worst pain, least pain, pain right now) and interference; Pediatric Pain Questionnaire (PPQ; pain right now, worst pain, and average pain items); Clinical Global Impression of severity, overall (CGI-severity: overall) scale; Clinical Global Impression of severity for mental illness (CGI-severity: mental illness) scale; Functional Disability Inventory-child version scale (FDI-child); Functional Disability Inventory-parent version scale (FDI-parent); Children’s Depression Inventory; Multidimensional Anxiety Scale for Children; and the percentage of patients with ≥30% and ≥50% reduction in the BPI average pain severity (response to treatment). Safety was reported based on AEs, laboratory values, height, weight, vital signs, and electrocardiograms. Suicide-related thoughts and behaviors were prospectively assessed by the Columbia-Suicide Severity Rating Scale (C-SSRS). AEs were classified based on the Medical Dictionary for Regulatory Activities version 20.1. A treatment-emergent adverse event (TEAE) was an event that first occurred or worsened in severity after baseline, on or before the last day, within the double-blind treatment period. The lowest level terms have been used for the TEAE computation and preferred terms are presented.

### Statistical analysis

The primary endpoint of the mean change in 24-h average pain severity of BPI [8] from baseline to Week 13 was analyzed based on a mixed-effects model repeated measures analysis using all the longitudinal observations at each post-baseline visit. Significant level was 0.05 with two-sided test. No adjustment was made for multiple comparisons. The model included the fixed categorical effects of treatment, pooled investigative site, week, and treatment-by-week interaction, as well as the continuous, fixed covariates of baseline value and baseline by week interaction. For all other continuous efficacy and safety variables, the last-observation-carried-forward (LOCF) approach was applied as appropriate; the last observation was defined as the last nonmissing observation obtained from Week 1 to Week 13. An analysis of variance (ANOVA) model with the main effects of treatment and pooled investigative site, or analysis of covariance (ANCOVA) with baseline values added as covariates, was used. The significance of treatment-by-pooled investigative site interaction was evaluated in a separate model, when appropriate. Type III sum-of-squares for the least squares (LS) means were used for the statistical comparison of main effects using ANOVA or ANCOVA. No statistical comparisons were conducted to compare the two treatment groups (placebo/duloxetine and duloxetine/duloxetine) during the open-label extension phase. Categorical comparisons between treatment groups were performed using Cochran-Mantel-Haenszel tests controlling for pooled investigative site and Fisher’s exact test for double-blind acute treatment phase, where appropriate. SAS® software v 9.4 was used for all analyses.

## Results

**Fig. 2:**
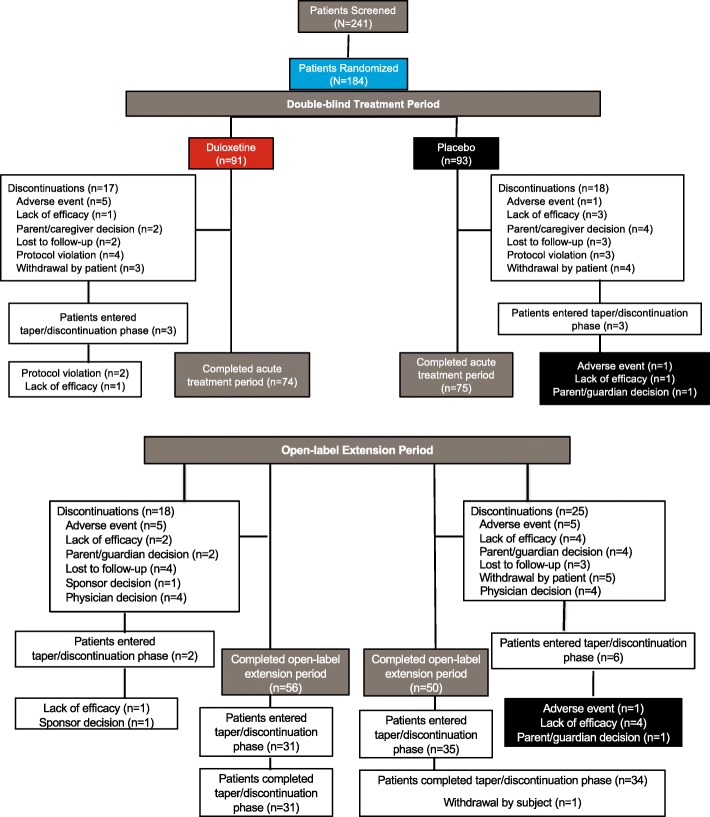
Patient Disposition

A total of 184 patients were randomized to duloxetine (*n* = 91) or placebo (*n* = 93), of which 149 (81.0%) patients completed the 13-week double-blind treatment period and 35 (19%) patients discontinued the study due to AEs (*n* = 6; 3.3%), lack of efficacy (*n* = 4; 2.2%), lost to follow-up (*n* = 5, 2.7%), protocol violation (*n* = 7, 3.8%), parent’s/caregiver’s decision (n = 6, 3.3%), or study withdrawal (n = 7, 3.8%) (Fig. 2). Furthermore, of 149 patients who completed the double-blind treatment period, 106 (71.14%) completed the open-label extension phase. The most frequently reported reasons for discontinuation during the open-label extension phase were withdrawal by subject and AEs (Fig. 2).

Overall, the patient demographics and baseline characteristics were similar between the treatment groups. The majority of the patients were Caucasian (77.2%, *n* = 142) and females (75.0%, *n* = 138) with a mean age of 15.53 years (Table 1). Between duloxetine and placebo groups, 11 (12.1%) and 7 (7.5%) patients received psychotherapy, 5 (5.5%) and 5 (5.4%) received physical therapy, and 79 (86.8%) 71 (76.3%) received other medications for any reason, respectively; however with no statistically significant difference between the treatment groups.

**Table 1:** Patients demographics and baseline characteristics (ITT population)

	Placebo (*n* = 93)	Duloxetine (*n* = 91)
Age, (years)	15.33 (1.42)	15.74 (1.38)
Gender, n (%)		
Female	65 (69.89)	73 (80.22)
Race, n (%)		
Caucasian	70 (75.27)	72 (79.12)
African American	8 (8.60)	7 (7.69)
Asian	7 (7.53)	6 (6.59)
American Indian or Alaska	0	1 (1.10)
Native Hawaiian or Others	1 (1.08)	0
Multiracial	6 (6.45)	4 (4.40)
BMI, kg/m^2^	24.10 (5.92)	24.11 (7.09)
Country, n (%)		
Argentina	19 (20.43)	19 (20.88)
India	7 (7.53)	6 (6.59)
Puerto Rico	3 (3.23)	2 (2.20)
United States	64 (68.82)	64 (70.33)
Time since diagnosis for fibromyalgia, years mean (SD)	0.38 (1.24)	0.19 (0.47)
BPI Average Pain, (0–10)		
Average Pain Score	5.63 (1.531)	5.68 (1.365)
worst pain score	6.87 (1.889)	7.11 (1.835)
least pain score	3.84 (1.974)	3.67 (2.108)
Pain score right now	5.19 (2.295)	5.18 (2.488)
BPI interference		
General activity	5.11 (2.343)	5.23 (2.362)
Mood	5.08 (2.638)	4.89 (2.639)
Walking ability	4.55 (2.819)	4.16 (2.857)
Normal work	4.90 (2.533)	4.65 (2.614)
Relations with other people	3.73 (2.840)	3.71 (2.778)
Sleep	5.60 (3.047)	5.89 (2.877)
Enjoyment of life	4.29 (2.831)	4.04 (3.080)
School work	4.26 (3.240)	4.86 (3.046)
BPI average interference Score (0–10)	4.69 (2.186)	4.68 (2.194)
PPQ score (0–100)		
Average pain score	57.51 (21.867)	61.45 (19.832)
Worst pain score	74.43 (21.295)	77.57 (20.005)
Pain score right now	51.16 (25.831)	49.91 (27.285)
CGI-S Score (1–7)		
Overall	4.1 (0.8)	4.1 (0.9)
Mental illness	2.03 (1.146)	2.11 (1.224)
FDI total score (0–60)		
Child total score	21.93 (10.046)	23.45 (11.129)
Parent total score (0–60)	22.12 (10.710)	22.87 (11.485)
CDI total score (0–54)	12.97 (7.645)	13.56 (7.078)
MASC total score (0–117)	43.61 (18.797)	45.56 (16.818)
Current medical status, Yes, n (%)		
Major depressive disorder	15 (16.13)	17 (18.68)
Generalized anxiety disorder	6 (6.45)	10 (10.99)
Attention deficit disorder	7 (7.53)	4 (4.40)
Concurrent/ongoing Therapy for FM Symptoms		
Psychotherapy	7 (7.5)	12 (13.2)
Physical Therapy	4 (4.3)	7 (7.7)

Values are mean (SD), unless specified; *BMI* body mass index, *BPI* Brief Pain Inventory, *CDI* Children’s Depression Inventory, *CGI-S* Clinical Global Impression: Severity, *FDI* Functional Disability Inventory, *MASC* Multidimensional Anxiety for Children, *PPQ* Pediatric Pain Questionnaire, *SD* standard deviation, *n* number of patients. BPI, 0 (no pain) to 10 (pain as bad as one can imagine); PPQ, 0 (no hurting, no discomfort, no pain) to 100 (hurting a whole lot, very uncomfortable, severe pain); CGI-S, 1 (normal, not at all ill) to 7 (among the most extremely ill patients); FDI, higher the score, more physical trouble or difficulty the child has doing regular activities; CDI, higher the score, more severe the depression; MASC, higher the total score, more severe the anxiety

At Week 13 (Double-blind treatment period), the least square (LS) mean change (standard error [SE]) from baseline in BPI average pain severity (primary endpoint) was not statistically significantly different between duloxetine 30/60 mg and placebo (− 1.62 [0.247] vs. −0.97 [0.244]; LS mean difference − 0.65 [0.330]; *p* = .052). Additionally, in post-hoc subgroup analysis of final doses, duloxetine (30 and 60 mg) vs. placebo, there was no significant difference in mean change of BPI average pain severity between the two doses versus placebo (LS mean [SE]: 30 mg − 1.68 [0.450]; 60 mg − 1.38 [0.309]) vs. −1.07 [0.249]; *p* = .37). During the double-blind treatment period, the LS mean changes from baseline (LOCF analysis) in severity and interference scores were not statistically significantly different between duloxetine and placebo, except for general activity (*p* = .030) and relations with other people (*p* = .013). Other secondary endpoints analyzed were also not significant, except for the rates of reduction in the BPI average pain severity by ≥30% and ≥50%, comparing duloxetine versus placebo (Table 2).

**Table 2:** Secondary endpoints at Week 13 (double-blind treatment period; LOCF analysis)

	LS means for change from baseline (SE)						
	double-blind treatment period				Open-label treatment period		Acute + Open-label
	Placebo (*n* = 93)	Duloxetine (*n* = 91)	Treatment difference	*p*-value	Placebo/duloxetine (*n* = 75)	Duloxetine/duloxetine (*n* = 74)	All patients randomized to duloxetine (*n* = 90)^a^
BPI Severity and Interference Ratings							
Worst pain score	−0.96 (0.267)	−1.43 (0.275)	− 0.47	0.175	− 0.8 (0.256)**	− 0.65 (0.262)*	− 1.66 (0.342)**
Least pain score	− 0.60 (0.242)	− 0.96 (0.247)	− 0.35	0.255	− 0.45 (0.212)*	−0.29 (0.218)	− 0.86 (0.267)**
Pain score right now	− 1.17 (0.270)	− 1.44 (0.276)	− 0.26	0.446	− 0.29 (0.252)	− 0.38 (0.259)	−1.31 (0.312)**
Incidence of Treatment Response^b^, n (%)							
≥30% Reduction	33 (36.26)	47 (52.22)	–	0.032†	27 (37.50)	25 (36.23)	49 (54.44)
≥50% Reduction	22 (24.18)	36 (40.00)	–	0.029†	18 (25.00)	17 (24.64)	44 (48.89)
BPI interference							
General activity	−0.99 (0.273)	−1.75 (0.280)	− 0.76	< 0.030*	− 0.20 (0.229)	− 0.18 (0.233)	− 1.74 (0.316)**
Mood	− 1.54 (0.273)	− 1.97 (0.279)	−0.43	0.220	−0.25 (0.258)	− 0.15 (0.270)	−1.59 (0.330)**
Walking ability	−1.21 (0.266)	−1.20 (0.271)	0.01	0.966	−0.21 (0.253)	−0.24 (0.260)	− 0.92 (0.285)**
Normal work	−1.19 (0.281)	−1.41 (0.286)	− 0.22	0.545	−0.32 (0.226)	− 0.62 (0.231)**	− 1.69 (0.328)**
Relations with other people	− 1.07 (0.236)	− 1.83 (0.241)	− 0.77	0.013*	− 0.41 (0.222)	− 0.12 (0.229)	−1.53 (0.264)**
Sleep	−1.16 (0.344)	−1.28 (0.352)	−0.12	0.788	−0.54 (0.284)	− 0.63 (0.292)*	− 1.67 (0.417)**
Enjoyment of life	−1.51 (0.251)	− 1.83 (0.257)	−0.32	0.320	−0.26 (0.236)	− 0.25 (0.243)	− 1.76 (0.288)**
School work	− 1.00 (0.324)	−1.48 (0.330)	− 0.49	0.243	−0.06 (0.271)	− 0.59 (0.278)*	− 1.94 (0.362)**
BPI average interference score	− 1.21 (0.217)	− 1.60 (0.221)	−0.39	0.165	−0.32 (0.187)	− 0.30 (0.192)	−1.61 (0.264)**
PPQ score							
Average pain score	−9.41 (2.946)	−11.03 (2.982)	−1.62	0.669	−6.44 (3.296)	−10.65 (3.080)**	− 18.01 (3.523)**
Worst pain score	−8.46 (3.322)	−14.36 (3.367)	−5.90	0.169	−8.06 (3.677)*	−4.15 (3.427)	−15.96 (3.954)**
Pain score right now	−7.20 (3.065)	−8.99 (3.092)	−1.79	0.647	−6.34 (3.335)	−4.74 (3.075)	−8.40 (3.441)*
CGI-S: Score							
Overall	−0.66 (0.118)	−0.88 (0.121)	− 0.22	0.146	− 0.67 (0.121)**	−0.67 (0.125)**	−1.25 (0.145)**
Mental Illness	−0.15 (0.087)	−0.16 (0.089)	− 0.01	0.927	− 0.24 (0.101)*	−0.20 (0.104)	− 0.27 (0.127)*
FDI total score							
Child total score	−5.00 (1.021)	−3.97 (1.038)	1.03	0.431	−1.03 (1.267)	−1.71 (1.202)	−5.02 (1.176)**
Parent total score	−4.17 (1.139)	−3.25 (1.152)	0.92	0.529	−2.27 (1.327)	−3.49 (1.227)**	−5.36 (1.280)**
CDI total score	−2.45 (0.674)	−3.28 (0.682)	−0.83	0.335	−1.41 (0.681)*	−0.42 (0.703)	−3.36 (0.865)**
MASC total score	−4.99 (1.558)	−6.21 (1.575)	−1.21	0.540	−0.78 (1.432)	−0.55 (1.478)	−6.90 (2.299)**

†From the Cochran-Mantel-Haenszel test controlling for pooled investigator sites; *p ≤ .05; **p ≤ .01; *BPI* Brief Pain Inventory, *CDI* Children’s Depression Inventory, *CGI-S* Clinical Global Impression-Severity, *FDI* Functional Disability Inventory, *SE* standard error, *n* number of patients, *MASC* multidimensional anxiety scale for children, *PPQ* Pediatric Pain Questionnaire. ^a^All patients who were randomized to duloxetine and had at least one non-missing post-baseline observation. ^b^Defined as reduction in BPI average pain for open label and double blind+open label periods, only within group change was tested

At Week 39 (open-label extension phase), the LS mean change (SE) from baseline in BPI average pain score was statistically significant in patients who were on placebo during double-blind treatment period and switched to duloxetine in open-label extension phase (placebo/duloxetine: − 1.11 [0.259]; *p* < .001). Similar results were observed in duloxetine/duloxetine patients (− 0.57 [0.217]; p = .01). Additionally, in all patients randomized to duloxetine (Week 0–39), the LS mean change from baseline in BPI average pain score was statistically significant within the treatment group, and across acute and open-label extension phases (− 1.63 [0.297]; p < .001). Other secondary endpoints analyzed in patients on duloxetine/duloxetine, placebo/duloxetine during open-label extension phase and all patients randomized to duloxetine are shown in Table 2.

During the double-blind treatment period, the percentages of patients reporting at least 1 TEAE were 82.42% (*n* = 75) for duloxetine versus 62.37% (*n* = 58) for placebo (*p* = .003). The most frequently reported TEAEs for duloxetine versus placebo were nausea (25.27% vs. 15.05%), headache (14.29% vs. 10.75%), vomiting (15.38% vs. 5.38%), and decreased appetite (15.38% vs. 3.23%) (Table 3). AEs leading to discontinuations were 5.49% (n = 5) with duloxetine versus 1.08% (*n* = 1) with placebo (Table 3). Serious AEs (SAEs) were reported by two (2.2%; appendicitis [n = 1]; suicidal ideation [n = 1]) duloxetine-treated patients and zero placebo-treated patients. None of the SAEs reported were considered to be study drug-related and none have led to study discontinuation. There were no deaths reported during the study. There were no significant differences between groups in suicide-related behaviors or ideation, as measured by the C-SSRS. Patients with weight gain were statistically significantly more in placebo group versus duloxetine group (LS means change from baseline [SE] 0.75 [0. 272] vs. −0.53 [0. 280]; *p* < .001). There were no other statistically significant differences between placebo and duloxetine group for systolic blood pressure (LS means change from baseline [SE] 0.67 [0.916] vs 0.47 [0. 938]; *p* = .869), diastolic blood pressure (0.34 [0. 709] vs 0.19 [0.726]; *p* = .867), height [SE] 0.21 [0.128] vs. 0.14 [0.130]; *p* = .669], and pulse rate (2.33 [0.912] vs 4.32 [0.936]; *p* = .094). Overall there were no statistically significant differences between placebo and duloxetine group in laboratory values except for creatine phosphokinase (units/L) (n [%] 1 [1.15) vs 8 [9.09]; *p* = .034), and creatinine (mm/L) (7 [8.05] vs 1 [1.14]; p = .034). Overall safety at the end of open-label extension period is shown in Additional file 2: Table S2. During the double blind period, change of heart rate (HR), based on ECG readings, was significantly higher in the duloxetine group vs. placebo [LS mean (SE) increase of 3.9 (1.121) beats per minute- BPM vs. −0.08 BPM (1.117); *p* = .005]. Patients who received duloxetine in the double blind period, as well as open label extension did not have a further increase in HR during the open label period; patients who received placebo during the double blind period and duloxetine during the open label extension had a 3.88 BPM (SE = 1.522, *p* = .012) increase in HR. All other ECG parameters were not significantly different between duloxetine and placebo.

**Table 3:** Overall safety during double-blind treatment period (safety population)

	Placebo (*n* = 93) n (%)	Duloxetine (*n* = 91) n (%)
TEAEs≥1, total	58 (62.37)	75 (82.42)
Mild	25 (26.88)	34 (37.36)
Moderate	29 (31.18)	33 (36.26)
Severe	4 (4.30)	8 (8.79)
Most frequently reported TEAEs (≥10%)		
Nausea	14 (15.05)	23 (25.27)
Headache	10 (10.75)	13 (14.29)
Vomiting	5 (5.38)	14 (15.38)
Decreased appetite	3 (3.23)	14 (15.38)
Columbia Suicidal-Severity Rating Scale		
Suicidal ideation or behavior	3 (3.23)	6 (6.59)
Non-suicidal self-injurious behavior	1 (1.08)	0
Serious adverse events	0	2 (2.2)
Discontinuation due to adverse events	1 (1.08)	5 (5.49)
Diarrhea	1 (1.08)	0
Nausea	0	1 (1.1)
Somnolence	0	1 (1.1)
Anxiety	0	1 (1.1)
Depressed mood	0	1 (1.1)
Suicidal behavior	0	1 (1.1)
Death	0	0

*n* number of patients, *TEAE* treatment-emergent adverse events

## Discussion

In this Phase 3b, randomized, double-blind, placebo-controlled, multi-site study in adolescents (aged 13–17 years) with JFM, duloxetine did not statistically significantly improve the primary outcome of mean change in 24-h average pain severity of the BPI at the end of double-blind treatment period compared to placebo (Week 13). However, there were some notable results in some of the secondary endpoints. For example, the ≥30% and ≥50% responder analyses showed a statistically significant reduction in BPI average pain severity in patients treated with duloxetine compared to those on placebo. The majority of the other secondary outcomes, including BPI interference, PPQ, and CGI, showed improvements with duloxetine over placebo but they were not statistically significant.

In the current study population, while the mean change (SE) in BPI average pain score (primary outcome) was not achieved, the − 0.65 (0.330) LS mean change difference in BPI average pain score observed was in the range (− 0.49 to − 1.23) that was significant (Except for − 0.49, *p* = .053) between duloxetine 60 mg and placebo in adult patients with FM, across four studies [9–12]. However, in a study in adult patients comparing duloxetine 30 mg and placebo, the BPI-Modified Short Form average pain severity reduction was not statistically significant between the treatment groups [13]. Moreover, in the current study, patients in the duloxetine group were allowed on the lower 30 mg (*n* = 32) dose if there were any tolerability issues with the 60 mg dose. Therefore a post-hoc analysis was performed comparing the 30 mg group with the 60 mg group, which showed no statistically significant difference in the primary endpoint between the groups.

Recruiting pediatric patients with JFM in the current study was challenging; it took almost 7 years and significant recruitment efforts to enroll the 184 pediatric patients in the current study. Similar recruitment challenges were reported in other randomized controlled clinical trials in patients with JFM [14, 15], one of which was terminated early due to low enrollment [15] and the other completed trial also failed to meet the primary objective of significantly reducing pain severity [14]. The treatment difference in the current JFM trial (− 0.65) was similar to results of the other completed trial of pregabalin in JFM (− 0.66) [14]. As reported by Arnold LM and colleagues [14], a larger sample size may lead to a different outcome, including in this study, given the trend towards improvement in the primary efficacy outcome., [14, 15]. Notably, during the open-label extension phase of the current study, patients in both placebo/duloxetine and duloxetine/duloxetine groups showed a statistical significance in LS mean change difference in BPI average pain score from baseline. Additionally, in all patients randomized to duloxetine, there was a statistically significant improvement in the majority of the efficacy measures across acute and open label periods compared to baseline. Among the patients in the trial, 16.13% (*n* = 15) and 18.68% (*n* = 17) patients had MDD and 6.45% (*n* = 6) and 10.99% (*n* = 10) patients had generalized anxiety disorder (GAD) at baseline in placebo and duloxetine groups, respectively. There was no statistically significant difference in mean change of BPI average pain severity between patients with or without GAD or those with or without MDD (data not shown).

The safety profile of duloxetine observed in this study was similar to that observed in previous pediatric duloxetine trials of other indications [16, 17], as well as in duloxetine trials in adults with FM [9, 13]. Nausea, headache, vomiting, and decreased appetite were the most frequently reported AEs in the present study, which are similar to those reported previously in adult population with FM [18]. In the present study, the suicidal ideation events reported with duloxetine were not significantly different from placebo-treated patients. Similar results were reported previously, including the exposure-adjusted analysis of suicidal ideation events, which have not shown any significant difference between duloxetine and placebo [18]. Increase in pulse rate was not statistically significant by vital sign data but HR was statistically significantly increased with duloxetine (vs. placebo) on ECG reading. Of note, vital signs were captured at each visit whereas ECG was only obtained 5 times during the study.

Several limitations of this study should be considered. First, the Yunus and Masi criteria [7] used for the diagnosis of JFM in this study are the most commonly used criteria for JFM. However, the Yunus and Masi criteria were based on evaluation of only 33 patients and more study is needed to validate the criteria. Second, the study did not collect data on history of failures on non-drug modalities. Because this was a randomized, placebo-controlled study, the variability related to non-drug treatment was expected to be equally distributed between duloxetine and placebo groups. Third, the findings from this study are limited to adolescents with chronic musculoskeletal pain meeting the Yunus and Masi criteria for JFM and may not be generalizable to other populations using alternative criteria for JFM, or adolescents with other types of chronic non-inflammatory musculoskeletal pain. Finally, initially, 210 patients were planned to randomize (1:1) into duloxetine and placebo treatment groups with an assumption of 70% completion rate, which would provide at least 80% power to detect the treatment difference of 1.0 point with α = .05 by Week 13. However, the actual acute phase completion rate was higher (81%), as observed during periodic reviews. Therefore, the enrolment was stopped at 184 patients, which provided a power of 80%.

## Conclusions

The primary study outcome of the present study was not significant for duloxetine versus placebo in treating JFM. However, one of the secondary endpoint, response to the treatment (30 and 50% reduction in average pain severity) was significant in duloxetine-treated patients compared to placebo-treated patients. There were no new safety concerns related to duloxetine in the study population.

## Additional files

Additional file 1: **Table S1.** Complete list of inclusion and exclusion criteria. (DOCX 18 kb)

Additional file 2: **Table S2.** Overall safety at the end of open label extension period (safety population)**.** N, number of patients entered to open label period; n, number of patients with specific TEAE; TEAE, treatment-emergent adverse events; *The denominators in the computation of the percentages and the analysis included only females (number of females = 53). (DOCX 13 kb)

## Abbreviations

AE: Adverse Events
ANCOVA: Analysis of Covariance
ANOVA: Analysis of Variance
BPI: Brief Pain Inventory
CGI: Clinical Global Impression
C-SSRS: Columbia-Suicide Severity Rating Scale
DSM: Diagnostic and Statistical Manual of Mental Disorders
FDA: Food and Drug Administration
FDI: Functional Disability Inventory
FM: Fibromyalgia
JFM: Juvenile Fibromyalgia
LOCF: Last-Observation-Carried-Forward
LS: Least Squares
MDD: major depressive disorder
PPQ: Pediatric Pain Questionnaire
QD: once daily
SAE: Serious Adverse Event
SE: Standard Error
TEAE: Treatment-Emergent Adverse Event
US: United States

## References

[1] King S, Chambers CT, Huguet A, MacNevin RC, McGrath PJ, Parker L (2011). The epidemiology of chronic pain in children and adolescents revisited: a systematic review. Pain..

[2] Perquin CW, Hazebroek-Kampschreur AA, Hunfeld JA, Bohnen AM, van Suijlekom-Smit LW, Passchier J (2000). Pain in children and adolescents: a common experience. Pain..

[3] Kashikar-Zuck S, Ting TV (2014). Juvenile fibromyalgia: current status of research and future developments. Nat Rev Rheumatol.

[4] Gómez-de-Regil L, Álvarez-Nemegyei J (2016). Open access scientific evidence of cognitive behavioral therapy for patients with fibromyalgia. Actualidades en Psicología.

[5] Gedalia A, Garcia CO, Molina JF, Bradford NJ, Espinoza LR (2000). Fibromyalgia syndrome: experience in a pediatric rheumatology clinic. Clin Exp Rheumatol.

[6] Varni JW, Burwinkle TM, Limbers CA, Szer IS (2007). The PedsQL as a patient-reported outcome in children and adolescents with fibromyalgia: an analysis of OMERACT domains. Health Qual Life Outcomes.

[7] Yunus MB, Masi AT (1985). Juvenile primary fibromyalgia syndrome. A clinical study of thirty-three patients and matched normal controls. Arthritis Rheum.

[8] Cleeland CS, Ryan KM (1994). Pain assessment: global use of the brief pain inventory. Ann Acad Med Singap.

[9] Arnold LM, Lu Y, Crofford LJ, Wohlreich M, Detke MJ, Iyengar S (2004). A double-blind, multicenter trial comparing duloxetine with placebo in the treatment of fibromyalgia patients with or without major depressive disorder. Arthritis Rheum.

[10] Arnold LM, Rosen A, Pritchett YL, D'Souza DN, Goldstein DJ, Iyengar S (2005). A randomized, double-blind, placebo-controlled trial of duloxetine in the treatment of women with fibromyalgia with or without major depressive disorder. Pain..

[11] Russell IJ, Mease PJ, Smith TR, Kajdasz DK, Wohlreich MM, Detke MJ (2008). Efficacy and safety of duloxetine for treatment of fibromyalgia in patients with or without major depressive disorder: results from a 6-month, randomized, double-blind, placebo-controlled, fixed-dose trial. Pain..

[12] Chappell AS, Bradley LA, Wiltse C, Detke MJ, D'Souza DN, Spaeth M (2008). A six-month double-blind, placebo-controlled, randomized clinical trial of duloxetine for the treatment of fibromyalgia. Int J Gen Med.

[13] Arnold LM, Zhang S, Pangallo BA (2012). Efficacy and safety of duloxetine 30 mg/d in patients with fibromyalgia: a randomized, double-blind, placebo-controlled study. Clin J Pain.

[14] Arnold LM, Schikler KN, Bateman L, Khan T, Pauer L, Bhadra-Brown P (2016). Safety and efficacy of pregabalin in adolescents with fibromyalgia: a randomized, double-blind, placebo-controlled trial and a 6-month open-label extension study. Pediatr Rheumatol Online J..

[15] Arnold LM, Bateman L, Palmer RH, Lin Y (2015). Preliminary experience using milnacipran in patients with juvenile fibromyalgia: lessons from a clinical trial program. Pediatr Rheumatol Online J.

[16] Strawn JR, Prakash A, Zhang Q, Pangallo BA, Stroud CE, Cai N (2015). A randomized, placebo-controlled study of duloxetine for the treatment of children and adolescents with generalized anxiety disorder. J Am Acad Child Adolesc Psychiatry.

[17] Emslie GJ, Wells TG, Prakash A, Zhang Q, Pangallo BA, Bangs ME (2015). Acute and longer-term safety results from a pooled analysis of duloxetine studies for the treatment of children and adolescents with major depressive disorder. J Child Adolesc Psychopharmacol.

[18] Choy EH, Mease PJ, Kajdasz DK, Wohlreich MM, Crits-Christoph P, Walker DJ (2009). Safety and tolerability of duloxetine in the treatment of patients with fibromyalgia: pooled analysis of data from five clinical trials. Clin Rheumatol.

