# Supplementary material for: Efficacy and safety of duloxetine versus placebo in adolescents with juvenile fibromyalgia: results from a randomized controlled trial
Source: Pediatr Rheumatol Online J. 2019 May 28;17:27. doi: 10.1186/s12969-019-0325-6 (PMC6540374; doi:10.1186/s12969-019-0325-6)
Supplement: Supplementary file 1 — Table S1. Complete list of inclusion and exclusion criteria. (DOCX 18 kb) [file 12969_2019_325_MOESM1_ESM.docx]

### Inclusion Criteria

Patients were eligible to be included in the study only if they met **all** of the following criteria:

[1] Outpatient male or female aged 13 to 17 years at the time of screening. Patients could not have turned 18 before the date of Visit 1 completion.

[2] Met criteria for primary JPFS as defined by Yunus and Masi (1985).

[3] Had a score of ≥4 on BPI average pain severity (Item 3) at Visit 1 and Visit 2.

[4] Female patients must have had a negative serum pregnancy test during screening. Furthermore, female patients must have agreed to abstain from sexual activity or to use a reliable method of birth control as determined by the investigator during the study. Examples of reliable birth control methods included: the use of hormone-containing contraceptives (for a minimum of 1 month prior to study enrollment); a reliable barrier method of birth control (diaphragms with contraceptive jelly, cervical caps with contraceptive jelly, condoms with contraceptive foam, intrauterine devices); partner with vasectomy; or abstinence.

[5] Patient’s parent/legal representative and patient were judged to be reliable by the investigator to keep all appointments for clinical visits, tests, and procedures required by the protocol.

[6] Patient’s parent/legal representative and patient, if capable, must have had a degree of understanding such that they can communicate intelligently with the investigator and study coordinator.

[7] Patients must have been capable of swallowing investigational product whole (without, for example, chewing, crushing, dissolving, or dividing the investigational product).

[8] Patients must have had venous access sufficient to allow blood sampling and be compliant with blood draws as per the protocol.

#### Disease Diagnostic Criteria

Patients must have had a diagnosis of JPFS. The diagnosis of JPFS, as defined by Yunus and Masi (1985), required the following 3 criteria to be met:

1. generalized musculoskeletal aching at 3 or more sites for 3 or more months in the absence of an underlying condition
2. the presence of at least 3 or more of the following 10 features present: chronic anxiety or tension, fatigue, poor sleep, chronic headaches, irritable bowel syndrome, subjective soft tissue swelling, numbness, pain modulation by physical activities, pain modulation by weather factors, and pain modulation by anxiety/stress.
3. 5 or more typical tender points. Note that 4 tender points satisfied diagnostic criteria provided that the patient had 5 of the 10 features listed under Criterion 2 above.

### Exclusion Criteria

Patients were excluded from the study if they met **any** of the following criteria:

[9] Were children of investigator site personnel directly affiliated with this study and/or their immediate families. Immediate family was defined as a spouse, parent, child, or sibling, whether biological or legally adopted.

[10] Were children of Lilly employees or employees of the designated CRO assisting with the conduct of the study.

[11] Were enrolled in, or discontinued within the last 30 days from, a clinical trial involving an investigational drug or device or off-label use of a drug or device (other than the investigational product/device used in this study), or concurrently enrolled in any other type of medical research judged not to be scientifically or medically compatible with this study.

[12] Had previously completed or withdrawn after randomization from a study investigating duloxetine.

[13] Had a known hypersensitivity to duloxetine or any of the inactive ingredients, or have frequent or severe allergic reactions to multiple medications.

[14] Had been treated with duloxetine within the last 6 months.

[15] Were not likely to benefit from duloxetine treatment, in the opinion of the investigator or had had prior nonresponse or inadequate tolerance to duloxetine for any clinical use.

[16] Had pain symptoms related to traumatic injury, past surgery, structural bone or joint disease (such as bursitis, tendonitis), or regional pain syndrome that in the opinion of the investigator, would interfere with interpretation of outcome measures.

[17] Currently had evidence of rheumatologic disorder or had a current diagnosis of RA, inflammatory arthritis, or infectious arthritis, or an autoimmune disease (for example, systemic lupus erythematosus).

[18] Had a Diagnostic and Statistical Manual of Mental Disorders (DSM)-IV Axis I condition, currently or within the past year, except MDD and/or GAD, adjustment disorder or specific phobias with PI approval.

[19] Had a current secondary DSM-IV Axis I condition of attention-deficit/hyperactivity disorder that requires pharmacologic treatment.

[20] Had any ***lifetime*** DSM-IV Axis I diagnosis of psychosis, bipolar disorder, or schizoaffective disorder.

[21] Had any DSM-IV Axis II disorder which, in the judgment of the investigator, would interfere with protocol compliance.

[22] Had a history of substance abuse or dependence within the past 6 months, excluding nicotine and caffeine.

[23] Had a positive urine drug screen (UDS) for any substances of abuse or excluded medication. Note: If the patient had a positive UDS at Visit 1 for an excluded medication that may not have had an adequate washout period, a re-test was to be performed and evaluated prior to Visit 2.

[24] Had a family history of 1 or more first-degree relatives (parents or siblings) with diagnosed bipolar I disorder (assessed by family member interview).

[25] Had a significant suicide attempt within 1 year of Visit 1 or were currently at suicidal risk in the opinion of the investigator. Note: Suicidal risk assessment was to be facilitated by the Columbia-Suicide Severity Rating Scale (C‑SSRS). Patients answering “yes” to any of the questions about active suicidal ideation/intent/behaviors occurring within the past 6 months were to be excluded (C-SSRS Suicide Ideation section—Questions 4 and 5; C‑SSRS Suicidal Behavior section, any of the suicide behaviors questions).

[26] Had a body weight <20 kg at any screening period visit.

[27] Exclusion criterion [27] was deleted in the Clinical Protocol dated 24 April 2013.

[28] Had a history of seizure disorder (other than febrile seizures)

[29] Were taking any excluded medications (for example, stimulants, antidepressants) that could not be discontinued at Visit 1.

[30] Had had treatment with fluoxetine within 30 days prior to Visit 1.

[31] Had had treatment with a monoamine oxidase inhibitor (MAOI) within 14 days of Visit 1; or the potential need to use an MAOI during the study or within 5 days of discontinuation of investigational product.

[32] Had abnormal thyroid-stimulating hormone (TSH) concentrations. **Note:** Patients previously diagnosed with hypothyroidism, who had been treated on a stable dose of thyroid supplement for at least the past 1 month and had medically appropriate TSH concentrations (on replacement therapy the TSH value may be below the reference range), and were clinically euthyroid could have participated in the study.

[33] Had uncontrolled narrow-angle glaucoma.

[34] Had acute liver injury (such as hepatitis) or severe cirrhosis (Child-Pugh Class C).

[35] Had a serious or unstable medical illness, including any cardiovascular, hepatic, renal, respiratory, hematologic, endocrinologic, or neurologic disease, or clinically significant laboratory abnormality or electrocardiogram (ECG) result that was not stabilized or was anticipated, requiring hospitalization within 6 months, or would compromise participation in the study in the opinion of the investigator. A serious or unstable medical condition is one that in the judgment of the investigator, is likely to require intervention, hospitalization, or use of excluded medications during the course of the study.

[36] Exclusion criterion [36] was deleted in the Clinical Protocol dated 24 April 2013.

[37] Female patients who were either pregnant, nursing or have recently given birth.

#### Exclusion Criteria for Regions with Prevalence of Chronic Hepatitis B Virus

[38] History of hepatic dysfunction, current jaundice, or positive hepatitis B surface antigen (Dane particle) (HBsAg) or positive hepatitis C virus antibody (HCV-Ab). Note: only for regions as determined by the Center for Disease Control (CDC 2010) with a prevalence of chronic hepatitis B virus(HBV).

[39] Patients with an alanine transaminase (ALT) ≥2 times the upper limit of normal (ULN) based on central lab reference range.
