# Supplementary material for: Efficacy and safety of duloxetine versus placebo in adolescents with juvenile fibromyalgia: results from a randomized controlled trial
Source: Pediatr Rheumatol Online J. 2019 May 28;17:27. doi: 10.1186/s12969-019-0325-6 (PMC6540374; doi:10.1186/s12969-019-0325-6)
Supplement: Supplementary file 2 — Table S2. Overall safety at the end of open label extension period (safety population). N, number of patients entered to open label period; n, number of patients with specific TEAE; TEAE, treatment-emergent adverse events; *The denominators in the computation of the percentages and the analysis included only females (number of females = 53). (DOCX 13 kb) [file 12969_2019_325_MOESM2_ESM.docx]

|  | **Placebo/**  **Duloxetine (N=75)**  **n (%)** | **Duloxetine/**  **Duloxetine (N=74)**  **n (%)** |
| --- | --- | --- |
| TEAEs≥1, total | 54 (72.00) | 52 (70.27) |
| Most frequently reported TEAEs (≥10%) | | |
| Nausea | 22 (29.33) | 10 (13.51) |
| Decreased appetite | 8 (10.67) | 6 (8.11) |
| Vomiting | 8 (10.67) | 4 (5.41) |
| Columbia Suicidal-Severity Rating Scale | | |
| Suicidal ideation or behavior | 2 (2.70) | 5 (6.80) |
| Non-suicidal self-injurious behavior | 5 (6.70) | 0 |
| Serious adverse events | 3 (4.00) | 3 (4.05) |
| Suicide attempt | 0 | 2 (2.70) |
| Appendicitis | 0 | 1 (1.35) |
| Discontinuation due to adverse events | 5 (6.67) | 5 (6.76) |
| Vomiting | 0 | 1 (1.35) |
| Myositis | 0 | 1 (1.35) |
| Headache | 0 | 1 (1.35) |
| Major depression | 0 | 1 (1.35) |
| Irritability | 0 | 1 (1.35) |
| Nausea | 1 (1.33) | 0 |
| Generalised tonic-clonic seizure | 1 (1.33) | 0 |
| Pregnancy* | 1 (1.89) | 0 |
| Affective disorder | 1 (1.33) | 0 |
| Rash | 1 (1.33) | 0 |
| Death | 0 | 0 |

**Supplementary Table 2**
